# Supplementary material for: MetaRibo-Seq measures translation in microbiomes
Source: Nat Commun. 2020 Jun 29;11:3268. doi: 10.1038/s41467-020-17081-z (PMC7324362; doi:10.1038/s41467-020-17081-z)
Supplement: Supplementary file 10 — Supplementary Data 7 [file 41467_2020_17081_MOESM10_ESM.zip › File2/Confidence_High_Taxonomy/333880_out.krona.html]

Javascript must be enabled to view this page.

members
magnitude
magnitudeUnassigned
count
unassigned
taxon
rank

333880\_out

15

2
superkingdom
15

phylum
1239
15

15
186801
class

15
186802
order

31979
family
15

15
1485
genus

species
1262805
7

SRS013476\_contig\_number\_25695SRS013965\_contig\_number\_1166SRS014287\_contig\_number\_16544SRS021484\_contig\_number\_9633SRS049712\_contig\_number\_6419SRS078419\_contig\_number\_36830SRS148159\_contig\_number\_19520


SRS019068\_contig\_number\_79953
1
2293053
species

3

SRS019685\_contig\_number\_6861SRS023914\_contig\_number\_22579SRS147139\_contig\_number\_43517
species
2293032

species
2293051
4

SRS015431\_contig\_number\_54231SRS015663\_contig\_number\_10325SRS063985\_contig\_number\_19262SRS077552\_contig\_number\_17000
